# All-Carbon Electrode Consisting of Carbon Nanotubes on Graphite Foil for Flexible Electrochemical Applications

**DOI:** 10.3390/ma7031975

**Published:** 2014-03-07

**Authors:** Je-Hwang Ryu, Gi-Ja Lee, Wan-Sun Kim, Han-Eol Lim, Mallory Mativenga, Kyu-Chang Park, Hun-Kuk Park

**Affiliations:** 1 Department of Biomedical Engineering and Healthcare Industry Research Institute, Kyung Hee University, Seoul 130-701, Korea; E-Mails: jhryu@khu.ac.kr (J.-H.R.); gjlee@khu.ac.kr (G.-J.L.); wskim731@khu.ac.kr (W.-S.K.); 2 Department of Information Display and Advanced Display Research Center, Kyung Hee University, Seoul 130-701, Korea; E-Mails: haneol.lim@gmail.com (H.-E.L.); mallory@tftlcd.khu.ac.kr (M.M.); kyupark@khu.ac.kr (K.-C.P.)

**Keywords:** carbon nanotube, carbon electrode, electrochemical application

## Abstract

We demonstrate the fabrication of an all-carbon electrode by plasma-enhanced chemical vapor deposition for use in flexible electrochemical applications. The electrode is composed of vertically aligned carbon nanotubes that are grown directly on a flexible graphite foil. Being all-carbon, the simple fabrication process and the excellent electrochemical characteristics present an approach through which high-performance, highly-stable and cost-effective electrochemical applications can be achieved.

## 1. Introduction

Due to its ability to graphitize, and its presence in different types of powders, fibers, fabrics and other composites, carbon represents a very attractive material for electrochemical applications [1,2], especially biosensors and energy storage devices [3,4]. Carbon electrodes can offer various potential advantages compared to conventional electrodes such as platinum or gold. Being feasible to customized systems, they can be fabricated with great suppleness in various forms and dimensions [4].

Among carbon materials, carbon nanotubes (CNTs) have outstanding properties such as excellent electrical and thermal conductivity, and large surface area. They are attractive candidates for not only electrodes in electrochemical devices but also other applications such as wiring [5], heat dissipation [6], and field electron emitters [7]. Another interesting carbon material, graphite, has useful characteristics such as a relatively simple fabrication process, cost effectiveness and mechanical flexibility due to the sheet-like hexagonal lattice arrangement of its carbon atoms [8].

For proper functionality, regular electrochemical devices rely on electrodes with high effective surface area [9]. The combination of CNTs and graphite produces hybrid electrodes with superior characteristics of both materials. Compared to other electrodes, such superior characteristics will include a larger surface area as well as mechanical flexibility, higher sensitivity and significantly lighter weight. To achieve the large surface area, an optimized growth of vertically aligned CNTs is a key parameter. The advantage of vertically aligned CNTs over random CNTs such as the spaghetti-like structured CNTs [10], is that the number of vertically aligned CNTs that can be packed per unit area of the substrate—with access to the conductive surface area of the CNTs—is substantially large. Hence, several chemical vapor deposition methods have been developed to grow aligned multi-walled CNT and single-walled CNT forests, but, it is very difficult to directly grow the vertically aligned CNT on a flexible carbon substrate [11,12].

In this work, we developed a new conducting and flexible, all-carbon electrode consisting of CNTs on graphite foil with nano-scaled carbon structures for use in highly sensitive electrochemical devices. The study provides information on the growth mechanisms of the CNTs on a graphite substrate, including their ensemble structures. It reveals the nanostructures of the CNTs and their measured electrochemical and mechanical properties.

## 2. Experimental Section

### 2.1. Preparation of All-Carbon Electrode

**Figure 1.**
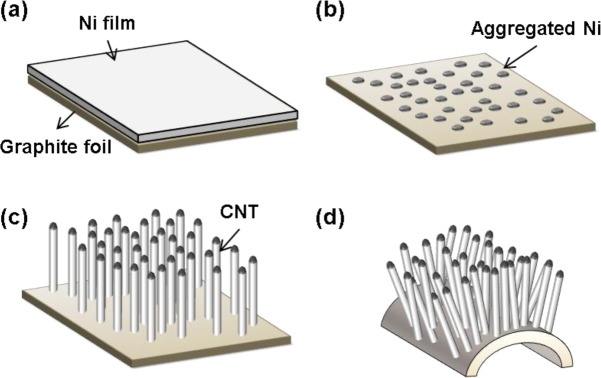
Fabrication and mechanical flexibility test of the all-carbon electrode: (**a**) Ni deposition on graphite foil; (**b**) annealing for seed formation; (**c**) carbon nanotube (CNT) growth and (**d**) flexibility test.

The fabrication of the flexible all-carbon electrode is depicted in Figure 1 Initially, a nickel catalyst film with 60-nm thickness was deposited on a 0.125-mm-thick graphite foil (Good fellow Corp., Coraopolis, PA, USA) by radio frequency (RF) magnetron sputtering using a nickel target of purity 99.9999% and 4-inches in diameter (Figure 1a). The graphite sample was positioned 10 cm above the target during sputtering. The seeding process was then initiated by forming at 600 °C for 30 min. The forming process induces the agglomeration of the Ni catalysts (Figure 1b).

The sample was then transferred to a plasma enhanced chemical vapor deposition (PECVD) system with a mesh grid placed 10 mm above the substrate holder for the growth of vertical CNTs [7]. The substrate electrode was maintained at −600 V with the top electrode grounded, and the spacing between the two electrodes was 20 mm in a triode-PECVD system. Acetylene (C_2_H_2_) and ammonia (NH_3_) gases were used for the CNT growth, with the C_2_H_2_:NH_3_ flow-rate ratio fixed at 40:60. The total gas pressure during growth was maintained at 2 Torr and the CNT growth time was 20 min. The growth temperature was maintained at 700 °C. The electric field direction during growth may provide the direction for the nanotube axes and the nanotube diameter can be controlled to a large extent by preparing catalyst particles of appropriate diameter (Figure 1c) [13]. The triode-PECVD system until now has been shown to produce aligned nanotubes, and can also be used to produce individual freestanding nanotubes, resulting in electrodes with a large surface area [14]. From the flexibility tests that were performed, the all-carbon electrode was found to be bendable (Figure 1d) and non-breakable. Three samples were processed in the triode-PECVD system for 3, 5 and 7 min in order to investigate the growth mechanism of the CNTs [15,16].

### 2.2. Electron Microscopy

Characterization of individual CNTs and catalysts at various growth stages, including high-resolution transmission electron microscope (TEM) imaging and scanning electron microscope imaging (SEM) was conducted using a FEI Tecnai F-30 (S) TEM with a field-emission electron gun operated at 300 keV and the Hitachi S-4700 high-resolution scanning electron microscope, respectively. For investigating microscopic interface between CNTs and graphite substrate, samples were prepared using a focused ion beam (FIB) (Quanta 3D FEG, FEI Company).

### 2.3. Electrochemical Measurements

Cyclic voltammetry (CV) experiments were performed using a PARSTAT 2263 advanced electrochemical analyzer (Princeton Applied Research, Oak Ridge, TN, USA) running Power CV software (Princeton Applied Research). All experiments were carried out by a three-electrode system with all-carbon electrodes as the working electrode, a platinum wire as the counter electrode, and an Ag/AgCl electrode as the reference electrode in DI water with the addition of 0.1 M potassium chloride (KCl) containing 1.0 mM potassium hexacyanoferrate(III) (K_3_Fe(CN)_6_, ACS reagent, Sigma-Aldrich, St. Louis, MO, USA). The CV measurement were recorded within a potential range from −0.1 to +0.5 V *vs*. Ag/AgCl.

## 3. Results and Discussion

### 3.1. Growth Mechanism of Carbon Electrode

**Figure 2.**
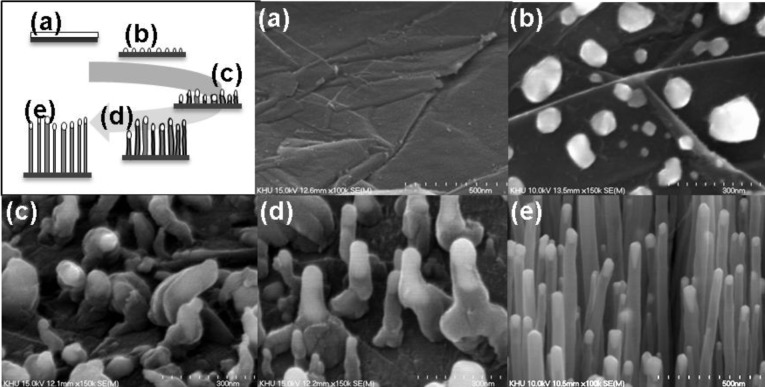
Schematic illustration and SEM images of growth mechanism of the all-carbon electrode. (**a**) bare graphite substrate; (**b**) aggregated Ni catalyst by forming process; (**c**) burgeon-like CNT at initial 3 min-CNT growth; (**d**) vertically shape of CNT after 5 min-growth; (**e**) vertically aligned CNT after 10 min.

A schematic illustration of the CNT growth on graphite foil is depicted in Figure 2. The surface of the graphite foil is not uniform but rather composed of stacked graphene layers (Figure 2a). The forming process at 600 °C for 30 min aggregates the Ni to form seed metals on the graphite foil (Figure 2b). The diameters of the seeds is 100~170 nm (average diameter: 140 nm). After 3 min into the growth process, burgeon-like structures of CNTs become visible (Figure 2c). The CNTs become further aligned with increasing growth time (Figure 2d). The CNTs took about 10 min to become vertically aligned (Figure 2e) [13].

### 3.2. Structural Properties of All-Carbon Electrode

**Figure 3.**
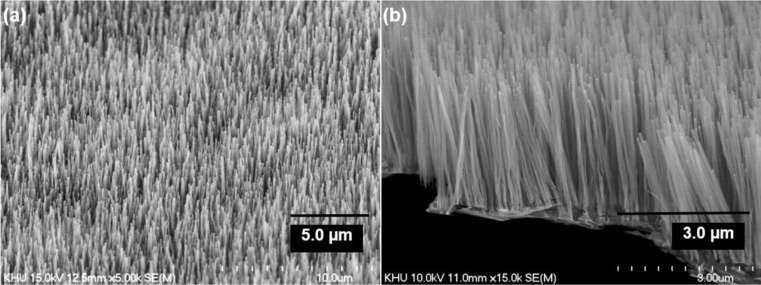
SEM image of (**a**) the morphology of all-carbon electrode; (**b**) cross-sectional SEM Image of the all-carbon electrode.

The all-carbon electrode demonstrated outstanding structural suppleness during mechanical bending. It could be bent to a radius of 1.5 cm without any visible cracks (Figure S1). The CNTs exhibit a very uniform forest-morphology (Figure 3a), which is one of the requirements for device applications. After growth, the CNTs are vertically aligned and approximately 3 μm in length. The diameter of the CNTs is 100 nm (Figure 3b). We can grow various nanostructures with nanofiber to nanotube diameters by controlling the thickness of the catalyst layer [17]. In this research, despite the large thickness of the CNTs, we can see a multilayered and hole structures from TEM images (See supplementary materials Figure S2). Uniform alignment leads to small sized substrates with larger surface areas. The result is outstanding electron transfer supporting capacity when used as electrodes in electrochemical reactions.

**Figure 4.**
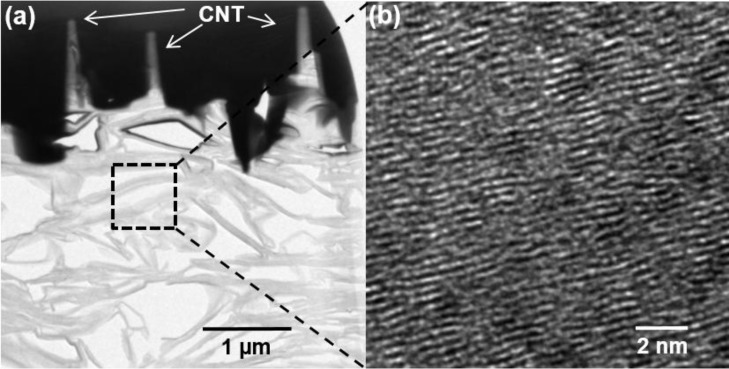
(**a**) Cross-sectional TEM image of the all carbon electrode consisting of CNTs and graphite substrate; (**b**) blow-up image of the area indicated by dashed rectangle.

The strong physical bond between the CNTs and the graphite substrate enables the structures to withstand mechanical stress during bending, making those applicable to flexible and non-breakable applications. The structure of the graphite foil is porous (Figure 4), which contributes to the flexibility of the graphite foil. As shown Figure 4b, the magnified substrate (Figure 4a) can see the crystalline graphite structure. The bond between the graphite substrate and CNTs is strong enough to withstand any mechanical stress. Although external force was used to detach the few CNTs from the sample for TEM analysis, the graphite is still firmly attached to the CNTs (Figure 4a) [18].

### 3.3. Flexibility Test

To quantitatively evaluate the influence of rolling on the electrical conductivity, the electrical resistances of the all-carbon electrode were measured under continuous rolling cycles, using a 6 × 6 cm^2^ flexible all-carbon substrate. The radius of curvature is 6 mm. Details of the rolling method can be found in the Figure S3 of supplementary materials.

**Figure 5.**
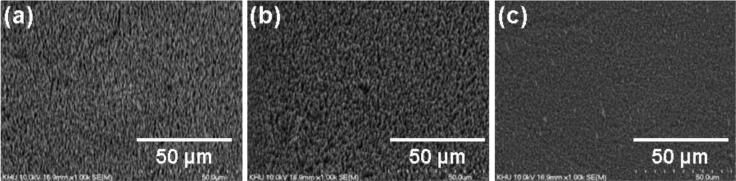
SEM images showing the surface morphology of the all-carbon electrode after the (**a**) 50th; (**b**) 150th; (**c**) 250th rolling cycle.

To investigate the reliability of the substrate, the morphology of the all-carbon electrode was traced with increasing number of rolling cycles and the results are summarized in Figure 5a–c. Regardless of the increasing bending stress, there is no cracking or peeling off of the CNTs from the graphite foil.

### 3.4. Electrochemical Characterization

To characterize the electrochemical behavior of all-carbon electrode after bending stress, we performed the CV measurements using standard electroactive reagent such as K_3_Fe(CN)_6_.

**Figure 6.**
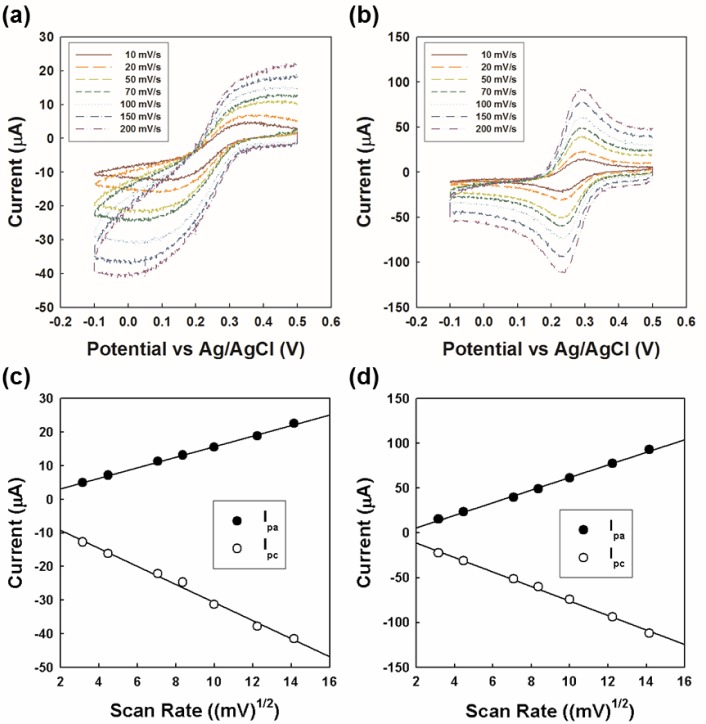
Cyclic voltammograms of (**a**) the graphite foil and (**b**) all-carbon electrode after 250th rolling cycles in 1.0 mM K_3_Fe(CN)_6_ in DI water with addition of 0.1 M KCl at potential scan rates ranging from 10 to 200 mV/s. The corresponding peak current dependence on the square root of scan rate in (**c**) the graphite foil and (**d**) all-carbon electrode after 250th rolling cycles.

Figure 6a,b represents the cyclic voltammograms of the graphite foil and all-carbon electrode after 250th rolling cycles in 1.0 mM K_3_Fe(CN)_6_ in DI water with addition of 0.1 M KCl at potential scan rates ranging from 10 to 200 mV/s. The corresponding peak current’s dependence on the square root of the scan rate is shown in Figure 6c,d. The anodic and the cathodic peak currents in both graphite foil and all-carbon electrode after 250th bending increased linearly with the square root of the scan rates (correlation coefficients of 0.9986 (anodic peak) and 0.9930 (cathodic peak) in graphite foil, and of 0.9980 (anodic peak) and 0.9971 (cathodic peak) in all-carbon electrode, respectively). The linear relationships indicate that the electrochemical kinetics reaction of both electrodes is diffusion-controlled [3]. However, the cyclic voltammograms of all-carbon electrode represented sharper and stronger peaks with a smaller peak separation than that of graphite foil. Potential separation of Fe(CN)_6_^3–/4–^ redox couple at a scan rate of 10 mV/s and the corresponding slopes of peak current *vs*. square root of scan rate are summarized in Table 1.

The potential separation (ΔE_p_) in all-carbon electrode between anodic peak potential (E_pa_) and cathodic peak potential (E_pc_) at 10 mV/s was 66.07 mV, which was more closer to the theoretical value of 57 mV for the reversible one-electron-transfer reaction at 25 °C than that of graphite foil (259.45 mV) [19]. Therefore, it suggests that the all-carbon electrode has better electrochemical reversibility than a graphite foil, indicating faster electron transfer capabilities at the electrode surface. Besides, the slope of the straight line of I_pa_ *vs*. v^1/2^ is 7.06 and 1.36 μA·mV^–1/2^·s^1/2^ in the all-carbon electrode and graphite foil, respectively. The slope of the I_pc_ *vs*. v^1/2^ is −8.10 and −2.67 μA·mV^−1/2^·s^1/2^ in the all-carbon electrode and graphite foil, respectively. Comparing the slopes of the curves, the kinetics of the electron transfer at the all-carbon electrode was improved, for both anodic and cathodic processes. It may be attributed to a larger surface area of the all-carbon electrode. Also, there is no influence of the all-carbon electrode with 250 and no rolling cycles (See supplementary materials Figure S4).

The calculated electroactive area of the all-carbon electrode was determined using the Randles-Sevcik equation for quasi-reversible electron transfer processes:

$$I_{p}=(2.65\times 10^{5})n^{\frac{3}{2}}ACD^{\frac{1}{2}}v^{\frac{1}{2}}$$ (1)

where *n* is the number of electrons participating in the redox process, *A* is the working electrode area (cm^2^), *D* is the diffusion coefficient (7.64 × 10^−6^ cm^2^·s^−1^ at 25 °C), *C* is the concentration of the probe molecule (1 × 10^−6^ mol·cm^−3^) and ν is the scan rate (V·s^−1^). From Equation (1), the electroactive area of the all-carbon electrode was 0.205 cm^2^, which was larger than that of graphite foil (0.067 cm^2^). Therefore, the excellent electrocatalytic performance of the all-carbon electrode can be attributed to the highly effective surface, which consists of large conducting regions.

## 4. Conclusions

An all-carbon electrode for use in electrochemical devices has been fabricated and investigated. The all-carbon electrode, consisting of CNTs grown directly on a flexible graphite foil, demonstrated a very large electrochemical active surface area and high electrocatalytic activity. Mechanical flexibility was confirmed, and the electrode exhibited reliable adhesion properties between the CNTs and graphite foil. The all-carbon electrode holds the promise of creating highly-stable, high performance, and cost-effective flexible electrochemical applications.

## Figures and Tables

**Table 1.** Electrochemical characterizations of graphite foil and all-carbon electrode after 250th bending which are calculated from cyclic voltammograms in the scan rate range 10~200 mV·s^−1^ using 1mM Fe(CN)_6_^3−/4−^ redox couple.

Parameters	Graphite foil	All-carbon electrode
ΔE_p_ (mV) *	259.45	66.07
Slope from I_pa_ *vs*. scan rate ^1/2^(μA·mV^−1/2^·s^1/2^)	1.36	7.06
Slope from I_pc_ *vs*. scan rate ^1/2^(μA·mV^−1/2^·s^1/2^)	−2.67	−8.10
Electroactive area, A_ea_ (cm^2^) **	0.067	0.205
Relative Roughness factor ***	1	3.05

* at a scan rate 10 mV/s;

** obtained value of slope from I_pa_ *vs*. scan rate ^1/2^;

*** A_ea_/A_ea-graphite_.

## Supplementary Materials

Supplementary File 1
