# Supplementary material for: All-Carbon Electrode Consisting of Carbon Nanotubes on Graphite Foil for Flexible Electrochemical Applications
Source: Materials (Basel). 2014 Mar 7;7(3):1975–83. doi: 10.3390/ma7031975 (PMC5453279; doi:10.3390/ma7031975)

# Supplementary Information

**Figure S1.** Optical image of a bended carbon electrode, indicating high mechanical flexibility and robustness of both sides of the electrode.

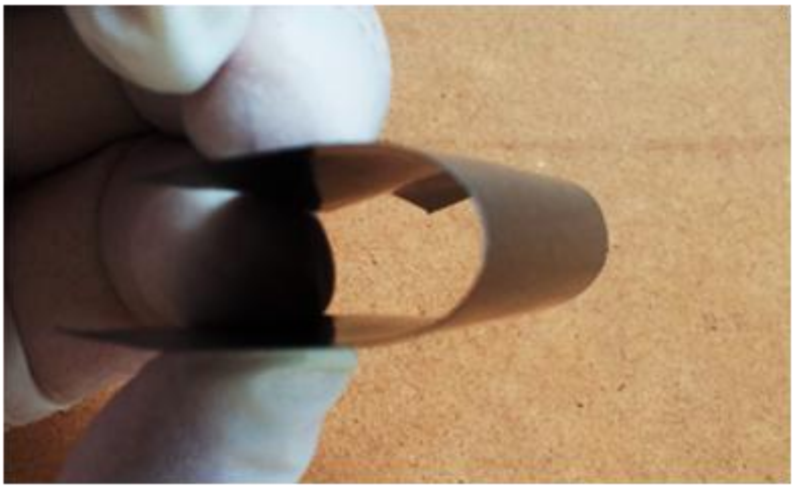

**Figure S2.** TEM image of tip area of CNT on all-carbon electrode.

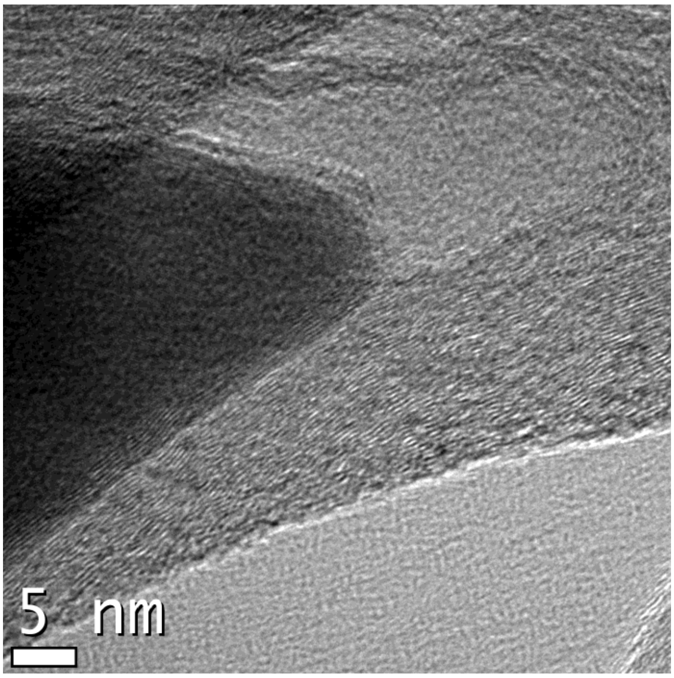


**Figure S3.** Rolling method of all-carbon electrode.

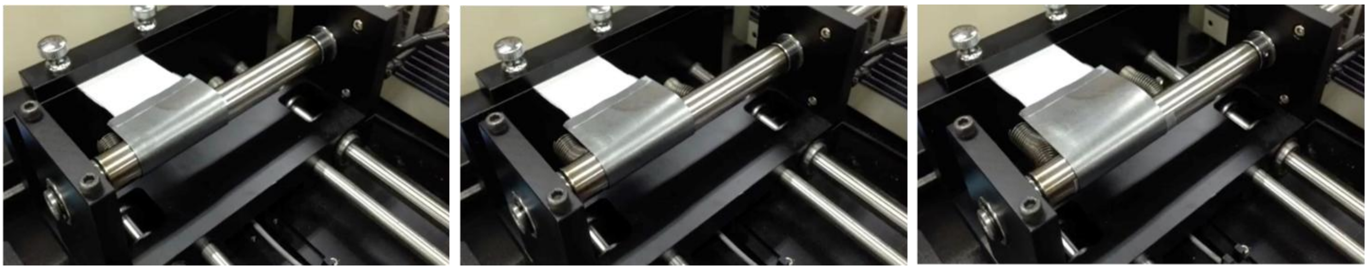

**Figure S4.** Cyclic voltammograms of (**a**) the graphite foil and (**b**) all-carbon electrode before rolling cycles in 1.0 mM $K_3Fe(CN)_6$ in DI water with addition of 0.1 M KCl at potential scan rates ranging from 10 to 200 mV/s. The corresponding peak current dependence on the square root of scan rate in (**c**) the graphite foil and (**d**) all-carbon electrode before rolling cycles.

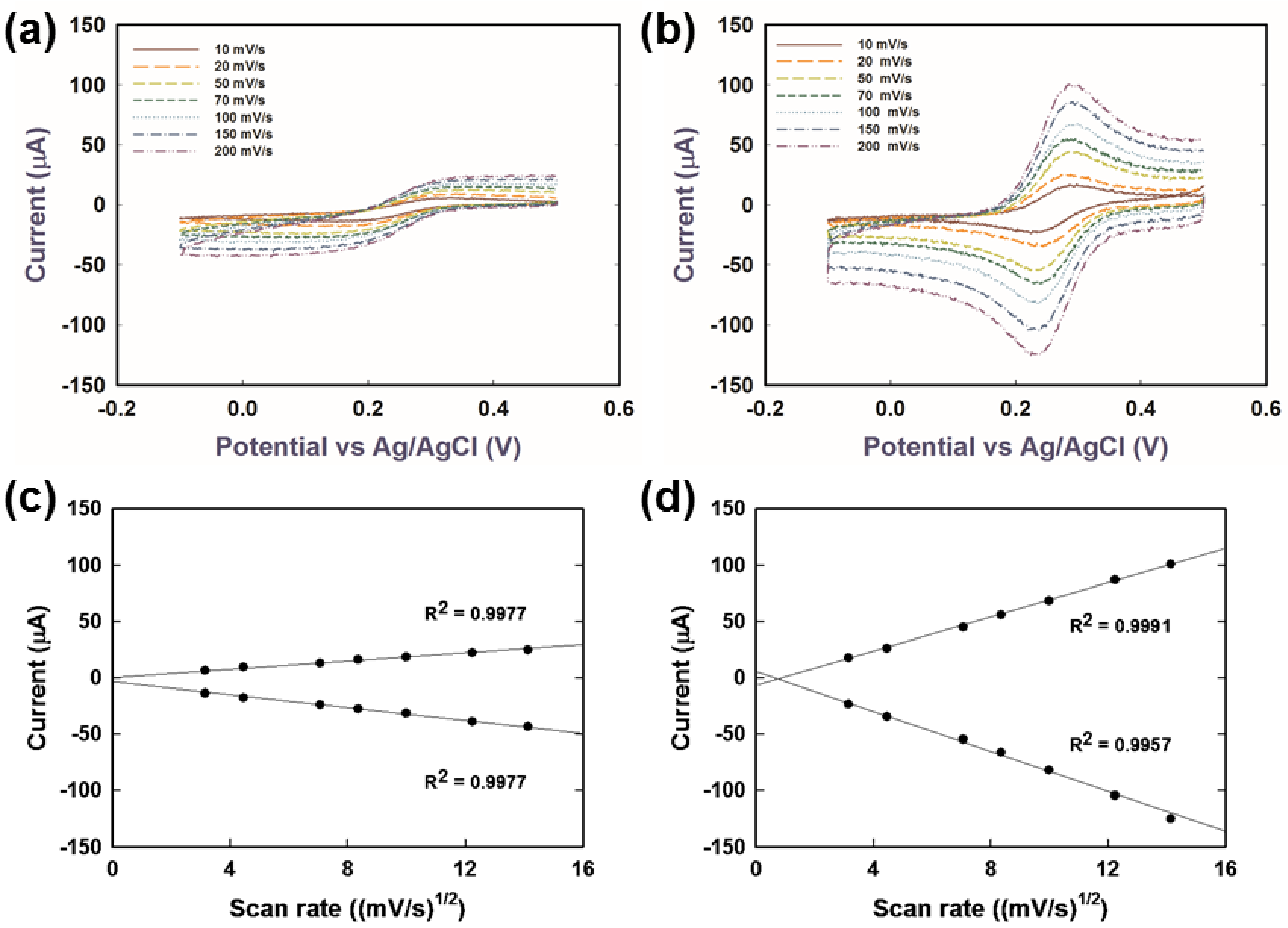

Supplement: Supplementary File 1 [file materials-07-01975-s001.pdf]
